# Supplementary material for: HIVprotI: an integrated web based platform for prediction and design of HIV proteins inhibitors
Source: J Cheminform. 2018 Mar 9;10:12. doi: 10.1186/s13321-018-0266-y (PMC5845081; doi:10.1186/s13321-018-0266-y)
Supplement: Supplementary file 2 — Additional file 2. Source code of HIVProtI web server. [file 13321_2018_266_MOESM2_ESM.zip › HIVprotI_Source-code/hivprotI/weka-3-6-6/doc/weka/gui/graphvisualizer/LayoutEngine.html]

LayoutEngine


---


|  |  |  |
| --- | --- | --- |
| **Overview** \| **Package** \| **Class** \| **Tree** \| **Deprecated** \| **Index** \| **Help** \| **Weka's home** | |  |
| **PREV CLASS**   **NEXT CLASS** | **FRAMES**    **NO FRAMES**     **All Classes** |  |
| SUMMARY: NESTED \| FIELD \| CONSTR \| METHOD | DETAIL: FIELD \| CONSTR \| METHOD |  |


---


## weka.gui.graphvisualizer Interface LayoutEngine

**All Known Implementing Classes:**: HierarchicalBCEngine

---

``` public interface LayoutEngine ```

This interface class has been added to facilitate the addition
of other layout engines to this package. Any class
that wants to lay out a graph should implement this
interface.

**Version:**
:   $Revision: 1.6 $ - 24 Apr 2003 - Initial version (Ashraf M. Kibriya)

**Author:**
:   Ashraf M. Kibriya (amk14@cs.waikato.ac.nz)

---

| **Method Summary** | |
| --- | --- |
| `void` | `addLayoutCompleteEventListener(LayoutCompleteEventListener e)`             This method adds a LayoutCompleteEventListener to the LayoutEngine. |
| `void` | `fireLayoutCompleteEvent(LayoutCompleteEvent e)`             This fires a LayoutCompleteEvent once a layout has been completed. |
| `javax.swing.JPanel` | `getControlPanel()`             This method returns the extra controls panel for the LayoutEngine, if there is any. |
| `FastVector` | `getNodes()`             give access to set of graph nodes |
| `javax.swing.JProgressBar` | `getProgressBar()`             This method returns the progress bar for the LayoutEngine, which shows the progress of the layout process, if it takes a while to layout the graph |
| `void` | `layoutGraph()`             This method lays out the graph for better visualization |
| `void` | `removeLayoutCompleteEventListener(LayoutCompleteEventListener e)`             This method removes a LayoutCompleteEventListener from the LayoutEngine. |
| `void` | `setNodesEdges(FastVector nodes, FastVector edges)`             This method sets the nodes and edges vectors of the LayoutEngine |
| `void` | `setNodeSize(int nodeWidth, int nodeHeight)`             This method sets the allowed size of the node |

**Method Detail**

### layoutGraph

```
void layoutGraph()
```

:   This method lays out the graph for better visualization

---


### setNodesEdges

```
void setNodesEdges(FastVector nodes,
                   FastVector edges)
```

:   This method sets the nodes and edges vectors of the LayoutEngine

---


### setNodeSize

```
void setNodeSize(int nodeWidth,
                 int nodeHeight)
```

:   This method sets the allowed size of the node

---


### getNodes

```
FastVector getNodes()
```

:   give access to set of graph nodes

---


### getControlPanel

```
javax.swing.JPanel getControlPanel()
```

:   This method returns the extra controls panel
    for the LayoutEngine, if there is any.

---


### getProgressBar

```
javax.swing.JProgressBar getProgressBar()
```

:   This method returns the progress bar
    for the LayoutEngine, which shows
    the progress of the layout process,
    if it takes a while to layout the
    graph

---


### addLayoutCompleteEventListener

```
void addLayoutCompleteEventListener(LayoutCompleteEventListener e)
```

:   This method adds a LayoutCompleteEventListener to the
    LayoutEngine.

    :   **Parameters:**: `e` - - The LayoutCompleteEventListener to add

---


### removeLayoutCompleteEventListener

```
void removeLayoutCompleteEventListener(LayoutCompleteEventListener e)
```

:   This method removes a LayoutCompleteEventListener from the
    LayoutEngine.

    :   **Parameters:**: `e` - - The LayoutCompleteEventListener to remove.

---


### fireLayoutCompleteEvent

```
void fireLayoutCompleteEvent(LayoutCompleteEvent e)
```

:   This fires a LayoutCompleteEvent once a layout has been completed.


---


|  |  |  |
| --- | --- | --- |
| **Overview** \| **Package** \| **Class** \| **Tree** \| **Deprecated** \| **Index** \| **Help** \| **Weka's home** | |  |
| **PREV CLASS**   **NEXT CLASS** | **FRAMES**    **NO FRAMES**     **All Classes** |  |
| SUMMARY: NESTED \| FIELD \| CONSTR \| METHOD | DETAIL: FIELD \| CONSTR \| METHOD |  |


---
